# The Application of Digital Volume Correlation (DVC) to Evaluate Strain Predictions Generated by Finite Element Models of the Osteoarthritic Humeral Head

**DOI:** 10.1007/s10439-020-02549-2

**Published:** 2020-06-22

**Authors:** Jonathan Kusins, Nikolas Knowles, Melanie Columbus, Sara Oliviero, Enrico Dall’Ara, George S. Athwal, Louis M. Ferreira

**Affiliations:** 1 Department of Mechanical and Materials Engineering, Western University, London, ON Canada; 2 Roth|McFarlane Hand and Upper Limb Centre, St. Joseph’s Health Care, London, ON Canada; 3 Department of Radiology, Cumming School of Medicine, University of Calgary, Calgary, AB Canada; 4 University of Calgary, Calgary, AB Canada; 5 Department of Oncology and Metabolism and INSIGNEO Institute for In Silico Medicine, University of Sheffield, Sheffield, UK

**Keywords:** Patient-specific finite element analysis, Digital volume correlation, Humerus FEM, CT-compatible loading, Osteoarthritis, Shoulder, Arthroplasty

## Abstract

Continuum-level finite element models (FEMs) of the humerus offer the ability to evaluate joint replacement designs preclinically; however, experimental validation of these models is critical to ensure accuracy. The objective of the current study was to quantify experimental full-field strain magnitudes within osteoarthritic (OA) humeral heads by combining mechanical loading with volumetric microCT imaging and digital volume correlation (DVC). The experimental data was used to evaluate the accuracy of corresponding FEMs. Six OA humeral head osteotomies were harvested from patients being treated with total shoulder arthroplasty and mechanical testing was performed within a microCT scanner. MicroCT images (33.5 *µ*m isotropic voxels) were obtained in a pre- and post-loaded state and BoneDVC was used to quantify full-field experimental strains (≈ 1 mm nodal spacing, accuracy = 351 *µ*strain, precision = 518 *µ*strain). Continuum-level FEMs with two types of boundary conditions (BCs) were simulated: DVC-driven and force-driven. Accuracy of the FEMs was found to be sensitive to the BC simulated with better agreement found with the use of DVC-driven BCs (slope = 0.83, *r*^2^ = 0.80) compared to force-driven BCs (slope = 0.22, *r*^2^ = 0.12). This study quantified mechanical strain distributions within OA trabecular bone and demonstrated the importance of BCs to ensure the accuracy of predictions generated by corresponding FEMs.

## Introduction

Bone tissue is a dynamic, continuously remodeling material that is sensitive to local mechanical stimuli.^15^ While remodeling is essential to ensure the bone’s structural integrity, pathologies such as osteoarthritis (OA) can result in abnormal remodeling which has the potential to compromise the overall function of the joint. In severe cases of functional loss or pain, the joint can be surgically treated by replacement with a prosthetic implant. Although bone is highly adaptable to local mechanical stimuli, differences in localized strains at the bone–implant interface compared to the native state can result in suboptimal bone adaptation that increases the risk of implant fixation failure.^17^,^27^ Therefore, to ensure the overall success rate of the joint replacement, it is important to consider how bone redistributes external loads on a local scale.

To predict localized strain within bone, specifically for shoulder joint arthroplasty, continuum-level patient-specific finite element models (FEMs) are often used which rely on density–modulus equations to assign linear isotropic material properties.^1^,^4^,^37^,^41^ These FEMs, combined with bone remodeling analytical algorithms,^10^ can be used to screen various joint replacement designs preclinically. However, before clinical adoption is feasible, experimental validation is critical to ensure the accuracy of displacements and strains predicted by these models. Recently, a round-robin study involving FEMs of the femur illustrated the impact of modelling assumptions, specifically the choice of material properties, on resultant strain predictions.^21^

To measure strain, strain gauges and digital image correlation (DIC) are a common surface-based measurement tool that provides an experimental benchmark for comparison to FEMs.^14^ While useful, these experimental measures on the surface offer no insight into the accuracy of strains predicted at the bone–implant interface, a critical region of interest for arthroplasty FEMs. To overcome this, digital volume correlation (DVC) has recently been proposed as an experimental measure that provides the capability to measure the resultant full-field strain within osseous specimens.^2^,^26^ Moreover, DVC has been applied at the whole bone level for the scapula,^36^,^42^,^43^ vertebra,^5^,^16^,^18^,^39^ and femur^29^,^32^ to further understand the internal deformations of bone. To the authors’ knowledge, DVC has yet to be applied to evaluate strain predictions generated by FEMs of the osteoarthritic humeral head, a growing field of interest as humeral head implants trend towards stemless designs.

The goal of the current study was to evaluate the accuracy of local strain predictions generated by continuum-level FEMs of the humeral head, through comparisons against experimental strains measured using DVC. There were two specific objectives: (1) combine mechanical loading with volumetric imaging to experimentally measure internal strains within OA humeral heads in a controlled experimental set-up; (2) replicate the experimental set-up with continuum-level patient specific FEMs to assess the accuracy of strain predictions compared to the experimental measures.

## Materials and Methods

A controlled experimental loading protocol was designed to induce localized load transfer in osteoarthritic (OA) trabecular bone at various locations with a pegged indenter, while obtaining micro computed tomography (CT) scans. Experimental trabecular strains were quantified using DVC. Continuum-level patient specific FEMs were generated for each humeral head to model the experimental conditions. Predicted strains from the FEMs were compared to the experimental strains.

### Specimen Acquisition and Experimental Testing

Six humeral head osteotomies were harvested from patients diagnosed with OA (Table 1) being treated with total shoulder arthroplasty (TSA) in accordance with Institutional Ethics (HSREB#113023). The superior direction of each humeral head osteotomy was marked at the time of surgery by a fellowship-trained surgeon. This direction was later confirmed on the corresponding clinical quantitative CT (QCT) scans following the registration process detailed in the “Finite Element Model Generation” section. The humeral heads were wrapped in phosphate-buffered saline (PBS) soaked gauze and stored at − 20 °C until testing.

**Table 1:** Patient demographics.

	Gender	Age (years)
Specimen 1	Female	62
Specimen 2	Male	76
Specimen 3	Female	54
Specimen 4	Female	59
Specimen 5	Male	68
Specimen 6	Male	82

**Figure 1:**
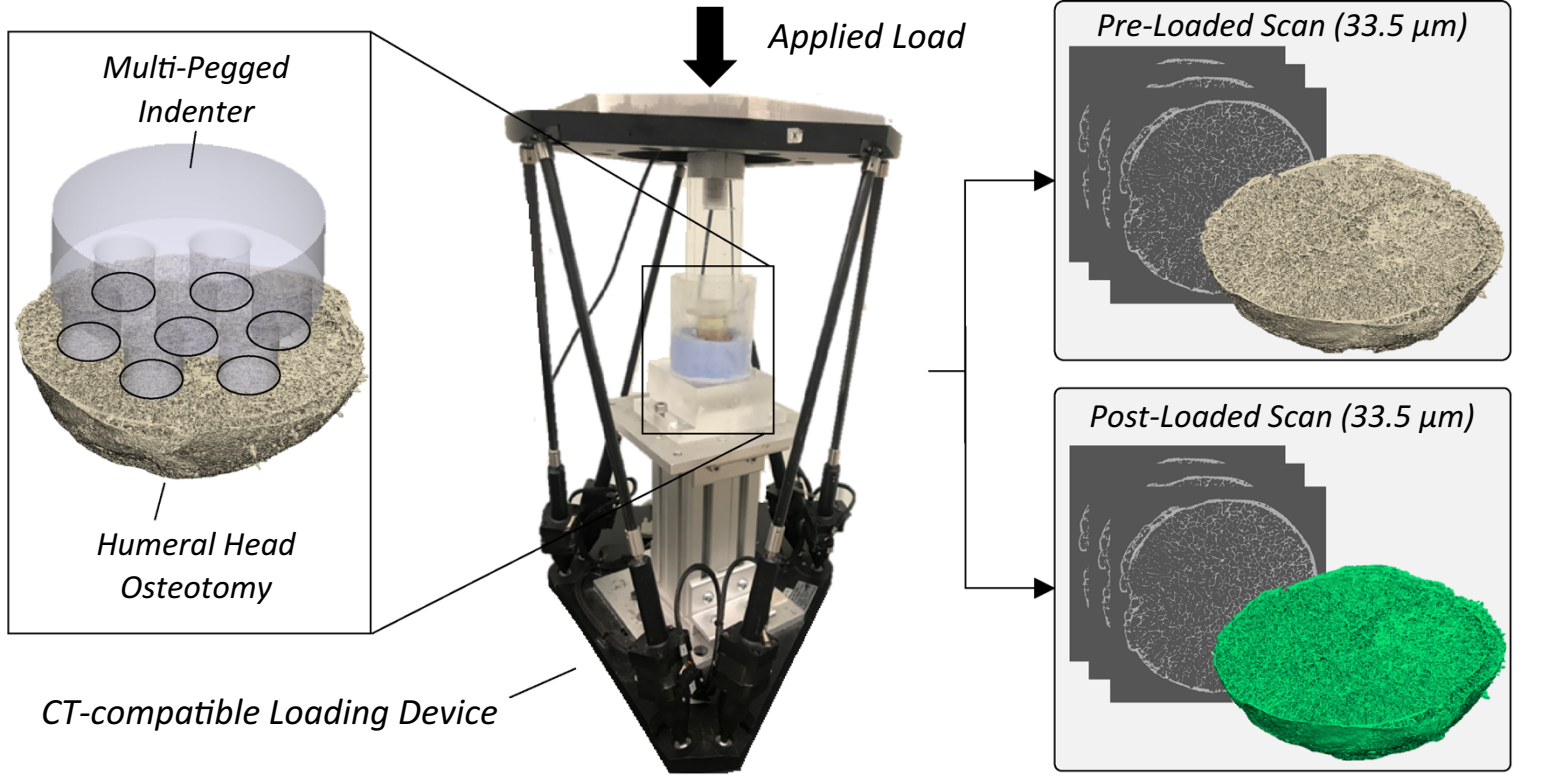
An acrylic indenter with seven pegs was used to load OA humeral head osteotomies (*n* = 6) within a microCT.

Prior to experimental testing, specimens were thawed for 1 h at room temperature in PBS solution. The articular surface of the humeral head osteotomy was potted in a silicone casting compound (durometer 65A) within an acrylic cylindrical tube (inner diameter = 76.2 mm, thickness = 3.2 mm) with the resection surface exposed. An additional custom fixture was used to ensure the plane of the resection surface was perpendicular to the axis of the cylindrical tube. After 1 h, the humeral head osteotomy was removed and refrozen. A simplified loading scenario was carried out which consisted of a custom fabricated indenter that was used to apply forces to the trabecular bone lying on the resection surface of the humeral osteotomy. A seven-pegged acrylic indenter (peg diameter = 7 mm) was fabricated with six peripheral pegs equally spaced at a diameter of 22.5 mm (Fig. 1). The multi-pegged indenter was designed to apply an external load to the trabecular bone at multiple locations and to mimic localized load concentrations of pegged glenoid implants. A previously reported CT-compatible loading device^22^,^25^ was used to apply compressive loads to the acrylic indenter within a cone-beam microCT scanner (Nikon XT H 225ST) and a 6-dof load cell (Mini 45, ATI Industrial Automation, NC, USA) measured the experimental loads applied by the loading apparatus. On testing day, each potted humeral head osteotomy was thawed for 2 h in PBS solution. After thawing, the acrylic tube containing the potted humeral head was centered within the loading device using a milled channel. Throughout the experimental loading and scanning protocol, the specimen was kept fully hydrated with PBS solution. The loading protocol began with a stabilizing load of 10 N while acquiring a pre-loaded microCT scan (33.5 *µ*m isotropic voxel size, 120 kVp, 110 *μ*A, 1571 projections, 2 frames per projection, 55-min scan time). Following, a post-loaded microCT scan was acquired with the specimen under a predefined applied load. The loading protocol consisted of applying a load of 500 N at a rate of 0.1 mm/s within the microCT scanner. A settling time of 20 min for tissue relaxation was allowed before acquiring a post-loaded scan. The resultant load was between 402 and 445 N for each specimen, measured immediately prior to the post-loaded scan. The resulting field of view (FOV) for each microCT scan (cube with edge lengths of 65 mm) was sufficient to capture the entire humeral head in both the pre- and post- loaded states.

### Trabecular Bone Strain Measurements

Local experimental trabecular bone strains between the pre- and post-loaded microCT images were obtained using a previously validated DVC algorithm (BoneDVC).^8^,^9^ Prior to performing the DVC analysis, pre-processing of the images was performed. First, a specimen-specific threshold was applied (Mimics v.20.0, Materialise, Leuven, BE) to generate a mask that contained only bone of the humeral head. Any values outside of the mask were assigned a constant grey level value (85 in 8-bit greyscale). The pre- and post-loaded images were then co-registered for each image set. Registration was performed (Amira 6.2.0, FEI Visualization Sciences Group, France) aligning the post-loaded image to the pre-loaded image using normalized mutual information as the optimization criterion. The normalized mutual information criterion uses the grey value histograms of each image and computes the joint entropy between them. The post-loaded image was then resampled using the resultant transformation matrix with Lanczos interpolator.^28^

To quantify full-field strain between the pre- and post-loaded images, BoneDVC was used. Details of the underlying algorithms have previously been reported.^8^,^9^ Briefly, BoneDVC is a global DVC registration technique that computes local displacements between two image sets. The displacements are then differentiated using finite element software (Ansys Mechanical APDL v.15.0, ANSYS, Inc., Canonsburg, USA) to calculate the full-field strain field between the pre- and post-loaded image sets. BoneDVC has previously been applied to validate full-field predictions of microCT- and QCT-based FEMs.^3^,^5^,^22^,^25^,^28^ To ensure the accuracy and precision of local strain measurements, a standard procedure of comparing two pre-loaded scans with various nodal spacing was performed.^8^ Based on these results, a nodal spacing of 30 voxels (sub-volume size of ≈ 1 mm) was determined as the optimal nodal spacing for the DVC registrations (mean average error for strain equal to 351 *µ*strain; standard deviation of the error for strain equal to 518 *µ*strain; precision for each components of displacement better than 2.79 *µ*m).

### Finite Element Model Generation

Continuum-level FEMs were generated from preoperative clinical QCT scans acquired for each patient (in plane pixel size = 0.55 to 0.65 mm; slice thickness = 1.25 mm). To identify the resection surface where the surgical osteotomy was made on the preoperative QCT scans, the corresponding pre-loaded microCT scans were used. Images generated from the QCT scans were registered to the coordinate system of the microCT using an iterative closest points algorithm (3-matic Research 11.0, Materialise, Leuven, BE) that aligned the outer geometry of the humeral heads derived from both scanners.^24^ The resection surface of the humeral head was then identified on the QCT scan and anything below the resection plane was removed by Boolean subtraction. A surface triangular mesh, edge length of 1 mm, was assigned to the virtual QCT humeral head osteotomy (3-matic v.12.0, Materialise, Leuven, BE). The mesh was then converted to a quadratic tetrahedral mesh using ABAQUS (v.6.14, Simulia, Providence, RI).

Linear isotropic elastic material properties were assigned in a similar manner as a prior experimentally validated humerus FEM^6^ that based its material properties on previous density–modulus relationships.^19^,^20^ A calibration phantom could not be included with patients within preoperative clinical QCT scans; therefore, a *post hoc* calibration equation was applied.^30^ This equation was obtained from six QCT scans that included a dipotassium phosphate (K_2_HPO_4_) calibration phantom (QCT Pro, Mindways Software, Inc., Austin, TX, USA) using the same clinical scanner at the same settings, while scanning a full cadaveric human arm. Local mechanical properties were assigned to the FEMs derived from the preoperative QCT scans based on Eqs. (1)–(3).

1 $$E_{\text{trab}} = 33,900\,*\,\rho_{\text{ash}}^{2.2} \;({\text{MPa}})\quad \rho_{\text{ash}} \le 0.3\,({\text{g}}/{\text{cm}}^{3} ),$$

2 $$E_{\text{trab}} = 2398\;({\text{MPa}})\quad 0.3 < \rho_{\text{ash}} < 0.486\,({\text{g}}/{\text{cm}}^{3} ),$$

3 $$E_{\text{cort}} = 10,200\,*\,\rho_{\text{ash}}^{2.01} \;({\text{MPa}})\quad \rho_{\text{ash}} \ge 0.486\,({\text{g}}/{\text{cm}}^{3} ),$$

where *E*_trab_ is Young’s modulus of trabecular bone, *E*_cort_ is Young’s modulus of cortical bone, and *ρ*_ash_ is ash density.

**Figure 2:**
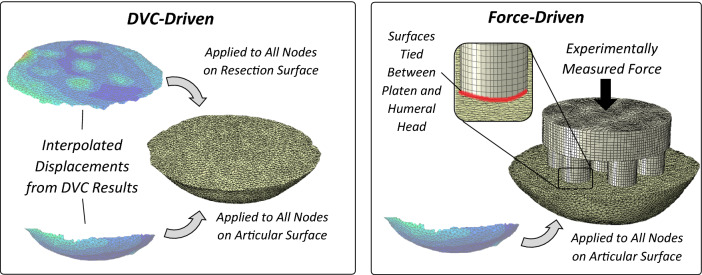
Finite element models were generated with two types of boundary conditions: DVC-driven and force-driven.

For each FEM model, two types of boundary conditions (BCs) were simulated: DVC-driven and force-driven (Fig. 2). DVC-driven BCs consisted of applying local experimental displacements to the nodes lying on the resection and articular surfaces of the humeral head osteotomy. The experimental displacements were extracted from the DVC measurements using custom-code (Matlab R2019a, MathWorks, Natick, USA) that used tri-linear interpolation to calculate the local displacements at the specified QCT-FEM nodes.^22^,^25^ For DVC-driven BCs, the predicted reaction force was calculated using the sum of local reaction forces computed at the nodes.^22^ Force-driven BCs were simulated by applying the experimentally measured force obtained by the load cell to a virtual loading platen that represented the multi-pegged indenter. The placement of the pegged indenter relative to the humeral head was ensured by registering the profile of the indenter to the pre-loaded microCT scan. A hexahedral mesh with homogenous material properties (*E* = 2960 MPa, *ν* = 0.37) were assigned to the virtual loading platen, and the surface between the virtual loading platen and humeral head resection surface were tied. To isolate load transfer at the peg–bone interface, experimental displacements were assigned to the articular surface of the humeral head consistent with the DVC-driven BCs (Fig. 2).

### Statistical Analysis

**Figure 3:**
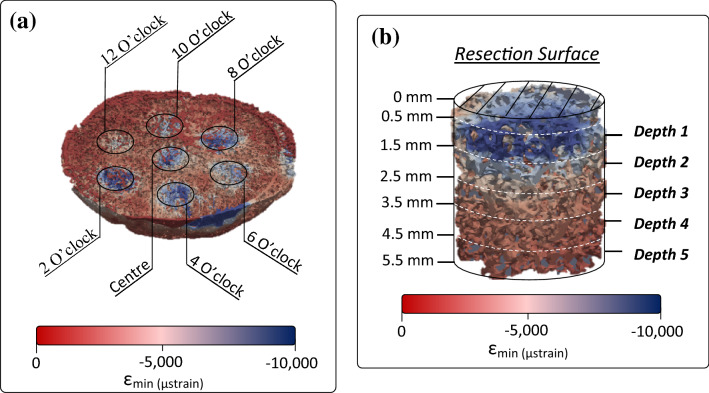
Regions of interest for analyzing full-field strains within the humeral head were divided based on peg position (**a**) and depth (**b**).

Experimental first (tensile) and third (compressive) principal strains were analyzed in varying regions of interest (ROI) that included peg position and depth from the resection surface. For each humeral head, seven cylindrical volumes of interest (7 mm diameter × 5 mm depth) were located 0.5 mm underneath each of the indenter’s pegs within the trabecular bone, in order to avoid the influence of DVC-driven boundary nodes. These cylindrical volumes of interest were further subdivided at 1 mm depths from the resection surface (Fig. 3b). In total, 35 ROIs were identified as a function of peg position and depth from the resection surface (Fig. 3). Within each ROI, first and third principal experimental strains were averaged for each specimen. To determine the influence of peg position and/or depth on the resultant strain measured, a 2-way repeated measures analysis of variance (ANOVA) was performed. Statistical significance was set at *p* < 0.05.

To determine the accuracy of strains predicted by the corresponding FEMs, third principal strains were averaged within each corresponding ROI for both force-driven and DVC-driven BCs. Linear regression was performed to analyze the agreement between the FEM predicted strains and the experimental measurements. Slope (*m*), *y*-intercept (*b*), and correlation coefficient (*r*^2^) were quantified for each humeral head specimen. The regression coefficients between the groups (DVC-driven and force-driven) were compared using an analysis of covariance (ANCOVA, Matlab v.R2019a, Natick, MA) with *α* = 0.05. A pooled linear regression was also completed which included all specimens and ROIs. A Bland–Altman analysis was performed to analyze the variance in agreement between the experimental and FEM results. To examine the influence of depth on the agreement between the FEM strain predictions and DVC results, root-mean-square error (RMSE) was calculated within each ROI. To pair the outcome measures, the FEM predicted strains were region averaged and paired with corresponding DVC strain measurements.^25^ RMSE% was calculated for each ROI by dividing the RMSE by the maximum strain value measured for each individual specimen (range of 15,768 to 19,025 *µ*strain). Finally, for FEMs with DVC-driven BCs, the percentage error associated with the predicted reaction force was calculated using the experimentally measured force as a reference measure.^22^.

## Results

### Experimental Trabecular Bone Strains

**Figure 4:**
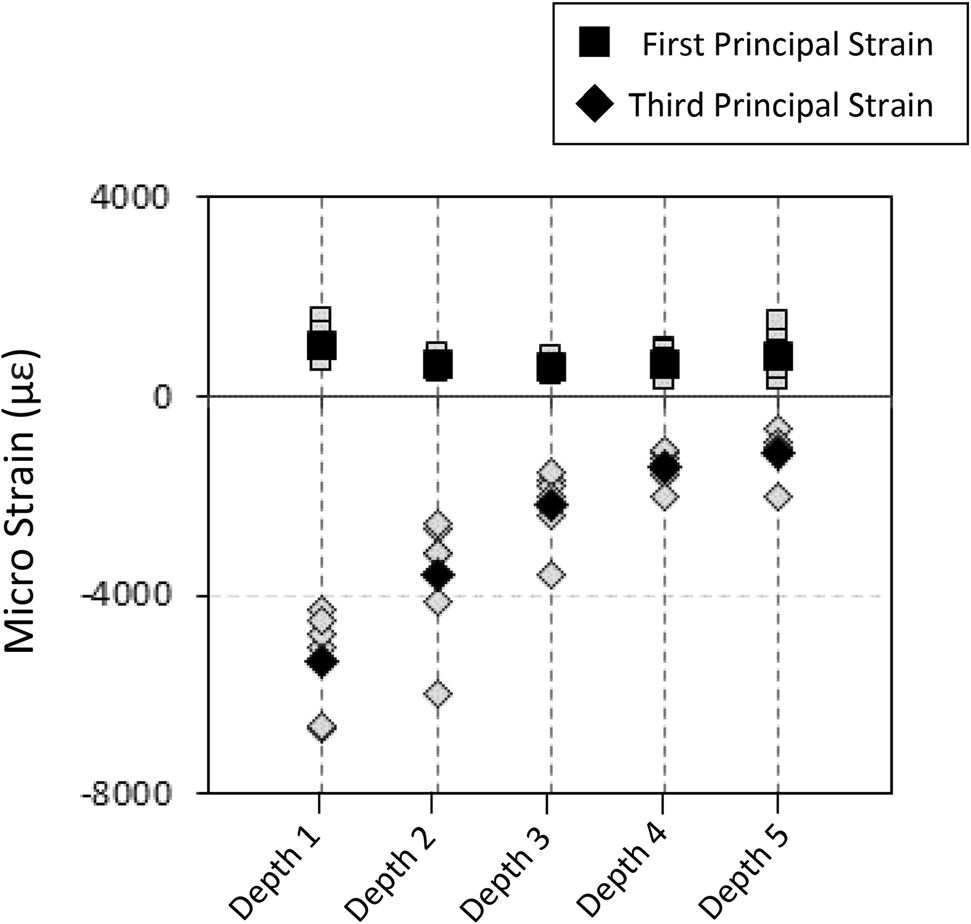
Experimental first (square) and third (diamond) principal strains averaged within each depth ROI defined from the resection surface. The average of all specimens (black) and specimen-specific strain (grey) are shown.

**Figure 5:**
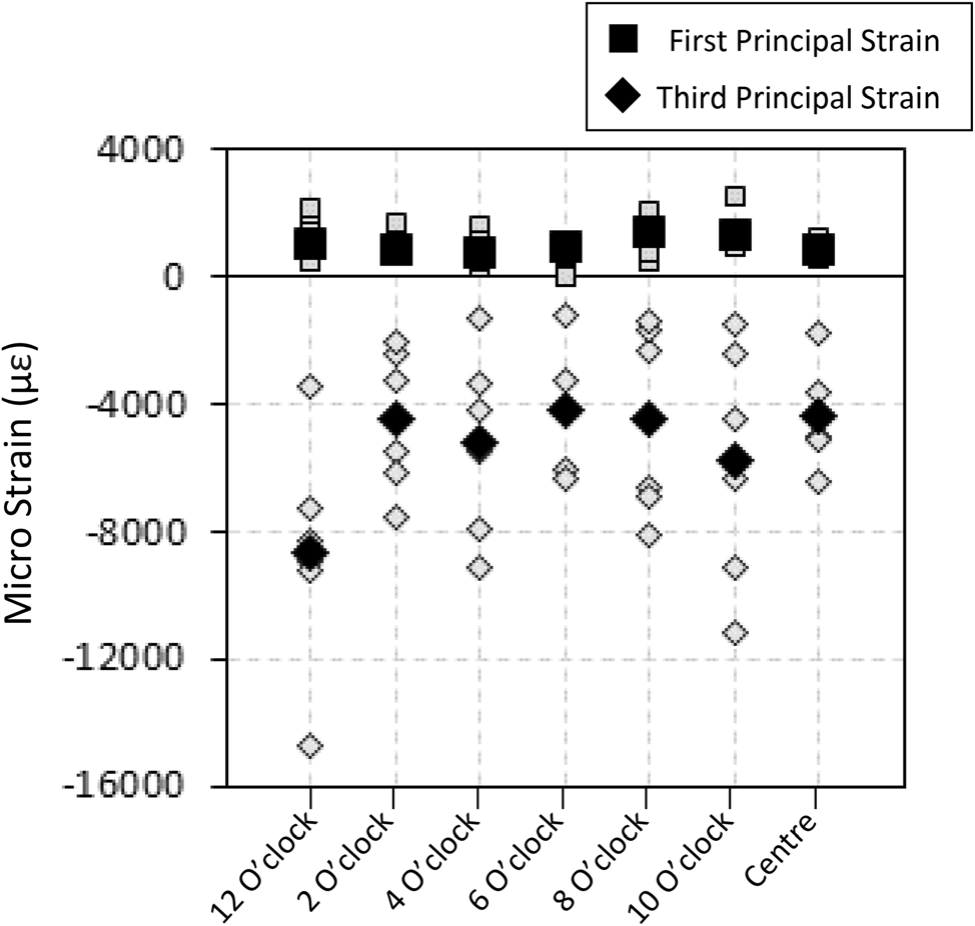
Experimental first (square) and third (diamond) principal strains averaged for each peg position within depth 1. The average of all specimens (black) and specimen-specific strain (grey) are shown.

Consistent for all humeral osteotomies, highest third-principal experimental strains were found at depth 1, the region closest to the indenter’s peg (Fig. 4). Depth from the indenter was found to have a statistically significant effect on the magnitude of third principal strain (*p* < 0.001) but not first principal strain (*p* = 0.183). Within depth 1, higher magnitude of strains were observed for third principal strain (mean = − 5322 *µ*strain, range − 4531 to − 6652 *µ*strain) compared to first principal strain (mean = 1042 *µ*strain, range 682 to 1587 *µ*strain). While the highest strains were observed underneath the 12 o’clock peg for four of the six specimens, peg position had no statistically significant effect on resultant third (*p* = 0.297) or first (*p* = 0.688) principal strains (Fig. 5).

### FEM vs. Experimental Comparison

Only third principal strains were compared between the FEM and DVC measurements due to the low magnitude of experimental first principal strains. Accuracy of the FEMs in predicting experimental strain was found to be sensitive to the BC simulated as indicated by the linear regression results (Table 2). Slope and *y*-intercept values improved (1:1 agreement indicated by slope = 1, *y*-intercept = 0) with the use of DVC-driven BCs (slope range = 0.68 to 1.02, *y*-intercept range = 243 to − 892) compared to force-driven BCs (slope range = 0.02 to 0.72, *y*-intercept range = − 582 to − 1920) and this was significant (*p* < 0.05) for five of the six specimens (Table 2). Strong correlations (0.81 < *r*^2^ < 0.94) were observed for all six specimens with the use of DVC-driven BCs; conversely, only weak to moderate correlations (0.02 < *r*^2^ < 0.62) were observed with the use of force-driven BCs.

**Table 2:** Specimen-specific regression results for FEM predictions of third principal strains.

Specimen #	Slope (*m*)			*y*-intercept, *b* (*µ*strain)			Coefficient of determination (*r*^2^)	
	Force-driven	DVC-driven	*p*-value	Force-driven	DVC-driven	*p*-value	Force-driven	DVC-driven
1	0.72	0.93	0.108	− 1136	− 892	0.618	0.62	0.82
2	0.08	0.75	< 0.001	− 1082	− 505	0.049	0.22	0.86
3	0.20	0.68	< 0.001	− 582	78	0.007	0.23	0.81
4	0.07	0.96	< 0.001	− 1081	243	0.003	0.06	0.84
5	0.45	1.02	< 0.001	− 1108	− 452	0.041	0.37	0.94
6	0.02	0.70	< 0.001	− 1920	− 764	0.004	0.02	0.87

**Figure 6:**
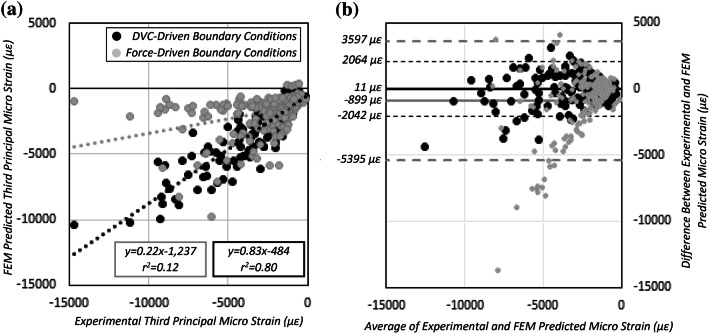
(**a**) Linear regression results between experimentally measured strains and FEM predicted strains with DVC-driven (black) and force-driven (grey) BCs. There were significant differences between BCs in slope (*p* < 0.001) and intercept (*p* < 0.001). (**b**) A Bland–Altman analysis of the error between FEM predictions and experimental strains for DVC-driven (black) and force-driven (grey) BCs.

When pooling the results of all specimens together, improvements in slope (*p* < 0.001), *y*-intercept (*p* < 0.001), and coefficient of determination were observed with DVC-driven BCs (*m* = 0.83, *b* = − 484, *r*^2^ = 0.80) compared to force-driven BCs (*m* = 0.22, *b* = − 1237, *r*^2^ = 0.12, Fig. 6). The Bland–Altman analysis indicated lower bias and tighter confidence intervals with the use of DVC-driven BCs (average error = 11 ± 2053 *µ*strain) compared to force-driven (average error = −899 ± 4496 *µ*strain) BCs (Fig. 6).

**Figure 7:**
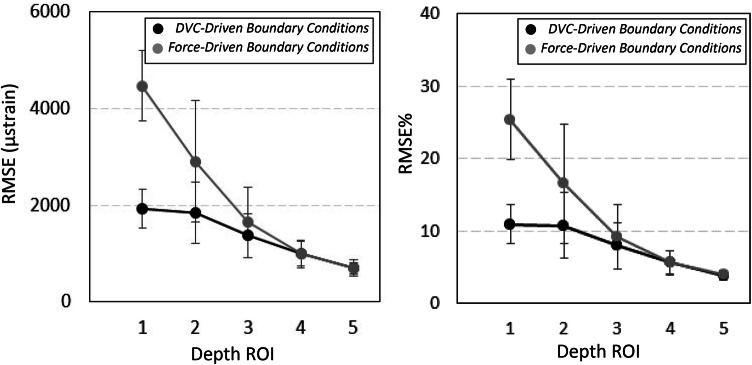
RMSE and RMSE% of FEM predictions with DVC-driven (black) or force-driven (grey) BCs compared to experimental third principal strain magnitudes.

RMSE was found to be highest within depth 1 with the use of force-driven BCs (RMSE = 4468 ± 723 *µ*strain, RMSE% = 25.4 ± 5.6%, Fig. 7). This error was reduced with the use of DVC-driven BCs at the same depth (RMSE = 1922 ± 400 *µ*strain, RMSE% = 10.9 ± 2.7%). RMSE% < 10% was observed in depth regions 3, 4 and 5 (average RMSE% < 10%) regardless of the BC simulated (Fig. 7).

The average predicted reaction force by the FEMs with DVC-driven BCs was 628 N (range 396 to 863 N) which corresponded to an average absolute percentage error of 47% (range 7 to 94%) when compared to the experimentally applied load.

## Discussion

The main goal of this work was to evaluate the accuracy of full-field strains predicted by continuum-level FEMs of the osteoarthritic humeral head. The prevalence of continuum-level FEMs continues to grow due to their ability to screen TSA implant designs preclinically^1^,^4^,^23^,^31^,^34^ but they rely on various modelling assumptions. Therefore, experimental validation is critical to ensure appropriate conclusions are drawn from these simulations. Within the current study, an experimental protocol that included microCT imaging of humeral head osteotomies under load combined with digital volume correlation (DVC) allowed for the validation of full-field strains predicted by corresponding FEMs.

The accuracy of our FEMs was found to be highly sensitive to the boundary condition (BC) simulated. For each specimen, improvement was observed with the use of DVC-driven BCs over force-driven BCs and this was also consistent for the pooled results (DVC-driven BCs: *m* = 0.83, *r*^2^ = 0.80, force-driven BCs: *m* = 0.22, *r*^2^ = 0.12). The reaction forces predicted by FEMs with DVC-driven BCs were relatively large [average percentage error = 47% (range 7 to 94%)]; however, these errors are in line with previous work that was conducted which used DVC-driven BCs within the shoulder.^22^ While improvements were observed with the use of DVC-driven BCs, a validated force-driven BC is desirable as it has the ability to extrapolate outside the experimental bounds. However, poor agreement within the current study highlights that further work is still required for experimental validation of these BCs. To generate the force-driven BCs, the surfaces between the pegged platen and trabecular bone were tied, which is a common modelling approach used in shoulder implant FEMs^1^,^4^,^35^; however, this may be an oversimplifying assumption that led to the observed poor agreement. While recent work has demonstrated the sensitivity that BCs have on whole-bone stiffness predictions of the femur,^33^ similar work has not investigated the dependence of localized strains on the BCs modelled for bone–implant constructs specifically for the shoulder. The results of this study highlight the importance of modelling these BCs accurately in order to obtain reliable simulation predictions.

The mechanical loading protocol within the current study relied on a simplified loading scenario which used a multi-pegged indenter to transfer load to the trabecular bone of the humeral head. As expected, highest experimental strains were observed at regions closest to the indenter within the trabecular bone. In addition, high variations in strain were observed underneath each individual peg at similar depths in the same specimen. Although peg position itself was found not to produce a statistically significant effect (*p* > 0.05) this may be attributed to the low sample size within the current study. Previous studies have shown that the stability of implant–bone interfaces within the shoulder can be influenced by local morphometric parameters^11^,^38^; therefore, it may also be conceivable that the resultant mechanical strain distribution observed within our results may be governed by similar parameters. While the use of a multi-pegged indenter is representative of glenoid implant designs and recent designs of stemless humeral implants,^13^ the lack of a single independent peg inhibited the ability to isolate and mechanically test local regions of trabecular bone. As well, the trabecular bone tested within the current study lies on the opposite side of the bone that receives the joint arthroplasty. However, this trade-off was accepted to allow for testing of patient-specific bone. Further mechanical testing of isolated trabecular bone cores, in combination with DVC and morphometric analyses, may elucidate optimal fixation strategies for future designs of glenoid or humeral implants.

The errors of our FEMs were found to be sensitive to the depth examined, with highest errors observed at the osteotomy resection plane. This coincided with the location of highest experimental strains at the platen–bone interface and is thus a critical region of interest to ensure the accuracy of predictions generated by FEMs. This highlights a strength of the current study, which is the use of DVC to quantify full-field strains immediately below the platen–bone interface that would otherwise not be attainable with surface-based measurement techniques (e.g. strain gauges or digital imaging correlation). Surface-based techniques may be more applicable for measuring cortical bone strains in fracture type scenarios^6^,^7^ but they are unable to resolve strain within the trabecular bone network. By measuring the internal strain throughout the bone, full-field predictions of the FEM predictions can be evaluated which is particularly relevant for applications interested in bone remodeling or fracture healing.^12^,^40^ Therefore, full-field experimental validation should be encouraged when attempting to examine the accuracy of FEM strains for bone–implant constructs.

This study had limitations. First, the FEMs of the humeral head were modelled using a linear isotropic material even though bone exhibits orthotropic material behavior. The use of isotropic material properties is commonly implemented by FEMs involving the humerus^6^,^7^; however, the effect of this assumption on local strain predictions should be further evaluated against full-field experimental measures for the humeral head. In addition, only one experimental load was applied within the elastic range of the humeral head; however, it is possible that local experimental yielding of trabeculae occurred. The developed experimental protocol could easily be adapted to examine fracture progression with stepwise loading; however, the poor agreement associated with our force-driven BCs makes it difficult to validate a non-linear FEM material model that includes fracture prediction or damage accumulation. Therefore, future work should aim to explore various methods in accurately modelling the load transfer between the loading platen and trabecular bone. As well, only first and third principal strains were examined within the current study; however, if non-linear loading is applied, additional strain metrics (*e.g.* von Mises strain) may become more prevalent. Finally, this study had a low sample size, a total of six humeral head osteotomies collected from patients undergoing TSA.

In conclusion, this study quantified mechanical strain distributions within OA trabecular bone at an osteotomized surface that is adjacent to the clinical bone–implant interface. Quantification of strains at this interface may be critical to ensure the longevity and success rate of joint replacement surgeries. The experimental data collected was used to evaluate the performance of corresponding CT-derived finite element models of the humeral head and elucidated the importance of modelling boundary conditions appropriately to ensure model accuracy.
